# Waning antibody responses in COVID-19: what can we learn from the analysis of other coronaviruses?

**DOI:** 10.1007/s15010-021-01664-z

**Published:** 2021-07-29

**Authors:** Ali Hamady, JinJu Lee, Zuzanna A. Loboda

**Affiliations:** Department of Immunology and Inflammation, Imperial College London, London, UK

**Keywords:** COVID-19, SARS, MERS, HCoV, Antibodies

## Abstract

**Objectives:**

The coronavirus disease 2019 (COVID-19), caused by the novel *betacoronavirus* severe acute respiratory syndrome 2 (SARS-CoV-2), was declared a pandemic in March 2020. Due to the continuing surge in incidence and mortality globally, determining whether protective, long-term immunity develops after initial infection or vaccination has become critical.

**Methods/Results:**

In this narrative review, we evaluate the latest understanding of antibody-mediated immunity to SARS-CoV-2 and to other coronaviruses (SARS-CoV, Middle East respiratory syndrome coronavirus and the four endemic human coronaviruses) in order to predict the consequences of antibody waning on long-term immunity against SARS-CoV-2. We summarise their antibody dynamics, including the potential effects of cross-reactivity and antibody waning on vaccination and other public health strategies. At present, based on our comparison with other coronaviruses we estimate that natural antibody-mediated protection for SARS-CoV-2 is likely to last for 1–2 years and therefore, if vaccine-induced antibodies follow a similar course, booster doses may be required. However, other factors such as memory B- and T-cells and new viral strains will also affect the duration of both natural and vaccine-mediated immunity.

**Conclusion:**

Overall, antibody titres required for protection are yet to be established and inaccuracies of serological methods may be affecting this. We expect that with standardisation of serological testing and studies with longer follow-up, the implications of antibody waning will become clearer.

## Introduction

Severe acute respiratory syndrome coronavirus 2 (SARS-CoV-2) was declared a pandemic in March 2020 by the World Health Organization. As of June 2021, it has caused over 3.8 million deaths and almost 180 million confirmed infections [1]. After intensive efforts from the scientific community over the past year, vaccines are now available. Almost all routine vaccinations rely on antibody responses [2], especially neutralising antibodies (nAbs), which are thought to be the best correlate of protection [3]. These can reduce infectivity by preventing attachment of the virion to the target cell, thereby blocking viral entry and therefore, replication [3].

Progression of some phase I/II trials of COVID-19 vaccines has depended solely on the magnitude of antibody response elicited, e.g. the immunogenicity endpoint of the BNT162b1 vaccine included nAb and receptor-binding domain (RBD)-binding IgG antibody titres [4] with no reports of B- or T-cell levels. However, recent studies have reported the rapid waning of antibodies following SARS-CoV-2 infection [5–7]. There is limited knowledge about the implications of this on long-term immunity, which is pertinent to address to ensure the success of public health strategies.

Seven coronaviruses are known to infect humans to date. Four of these are human coronaviruses (HCoVs) 229E, NL63, OC43 and HKU1, which cause relatively mild symptoms and circulate as endemic strains of the common cold. The other three, Middle East respiratory syndrome coronavirus (MERS-CoV), SARS-CoV and SARS-CoV-2 can cause life-threatening respiratory infections [8]. However, even the HCoVs may have started as more severe infections, e.g. OC43 has been stated as a possible aetiological agent for the “Russian flu” pandemic [9]. Their clinical and non-clinical characteristics have been summarised below (Table 1).

**Table 1:** A comparison of clinical and non-clinical characteristics of coronaviruses SARS-CoV, MERS-CoV, HCoVs and SARS-CoV-2 [1, 10–25]

	SARS-CoV	MERS-CoV	HCoVs (229E, OC43, NL63, HKU1)	SARS-CoV-2
Genus	*Betacoronavirus*	*Betacoronavirus*	*Alphacoronavirus*: **229E, NL63** *Betacoronavirus*: **HKU1, OC43**	*Betacoronavirus*
Spike similarity to SARS-CoV-2	77–97.71%	32.79%	**229E**: 30% **NL63**: 28% **OC43**: 31.26% **HKU1**: 30.5%	–
Host cellular receptor	ACE2	DPP-4	**229E**: APN **NL63**: ACE2 **OC43** and **HKU1**: 9-*O*-acetylated neuraminic acid	ACE2
Reservoir: intermediary host	Bat–civet	Bat–camel	**229E**: bat–camel **NL63**: bat–? **OC43**: mouse–cow **HKU1**: mouse–?	Bat–?
Mode of transmission	Respiratory droplet, close contact with infected individual, aerosol, possibly faecal–oral	Respiratory droplet, close contact with infected individual/camel, aerosol, consumption of unpasteurised camel milk	Respiratory droplet, close contact with infected individual, aerosol	Respiratory droplet, close contact with infected individual, aerosol, possibly faecal–oral
Emergence	February 2003	June 2012	**229E**: 1965 **OC43**: 1967 **NL63**: 2004 **HKU1**: 2005	December 2019
Current status^a^	Contained as of May 2004	Sporadic	Endemic	Pandemic
Infected cases^a^	> 8000	> 2500	N/A	> 178 million
Number of deaths^a^	> 770	> 880	N/A	> 3.8 million
Case fatality rate^a^	~ 10%	~ 34%	N/A	~ 2%
Risk factors for severe disease	Age > 60 years, comorbidities (heart disease and diabetes mellitus), elevated lactate dehydrogenase and neutrophil count at admission	Male sex, age ≥ 65 years, comorbidities, concomitant infection, low serum albumin (< 35 g/L)	Immunocompromise, age < 5 years and ≥ 65 years, respiratory co-infection	Male sex, age ≥ 60 years, non-white ethnicity, comorbidities, dyspnoea, haemostatic abnormalities, respiratory rate ≥ 24 breaths/min, SpO_2_ < 90% at admission
Clinical manifestations	Fever, headache, muscle aches, malaise, non-productive cough, dyspnoea, respiratory failure in 10–20% Mostly affected adults aged 25–70 years	Fever, cough, dyspnoea, pneumonia, vomiting or diarrhoea, fatigue, myalgia, respiratory failure. 21.5% of cases are mild/asymptomatic	Cause 20–30% of “common colds”, congestion, malaise, headache, fever, sore throat, 50–90% symptomatic. Severe causes: bronchiolitis, pneumonia, croup	Most develop mild–moderate severity illness. Fever, non-productive cough, fatigue, anosmia, dyspnoea, chest pain, pneumonia, respiratory failure, coagulopathy

*N*/*A* information not available, *SARS-CoV* severe acute respiratory syndrome coronavirus, *MERS-CoV* Middle East respiratory syndrome coronavirus, *HCoV* human coronavirus, *ACE2* angiotensin converting enzyme 2, *DPP-4* dipeptidyl peptidase-4

^a^MERS-CoV cases and deaths correct as of April 2021, and SARS-CoV-2 cases and deaths correct as of 20th June 2021

Coronaviruses are composed of four structural proteins including spike, envelope, membrane and nucleocapsid [13]. Cellular infection occurs when the RBD of the spike protein’s S1 subunit attaches to its host cellular receptor, causing a conformational change in the S2 subunit which mediates fusion and entry into the cell [26]. Antibodies to this spike protein have been shown to be most important in providing protective immunity in SARS-CoV [27]. Given the genetic homology and similarity in spike proteins between MERS-CoV, SARS-CoV and SARS-CoV-2, antibody responses to these viruses may demonstrate a certain degree of similarity and parallels may be drawn between their pathogenicity [12, 13].

This narrative review aims to compare the antibody responses to different human coronaviruses to further our understanding of long-term immunity in SARS-CoV-2. We critically summarise the evidence for the duration and efficacy of antibodies in protective immunity and explore the implications of antibody waning on public health strategies. We also discuss some links between antibody waning, cross-reactivity and vaccine efficacy which may be important in future research.

## Methods

After deciding the title and subtitles, we conducted searches on the PubMed and Embase databases up to June 20, 2021. Ahead-of-print publications and those on preprint servers were included given the fast developments in the COVID-19 pandemic. The search included keywords such as: “antibod*”, “seropositiv*”, “cross-reacti*” and “immun*” alongside “COVID-19” (OR “SARS-CoV-2”), “HCoV-*” (OR “seasonal coronavirus”), “MERS*” or “SARS-CoV” (OR “SARS”). Most articles retrieved were primary research papers. We also identified articles from the reference lists of other papers. We did not contact authors to obtain unpublished data. Figures of antibody kinetics are solely a graphical representation of the estimated trend in nAb titres based on severity and waning over time, generated using various studies.

## Antibody waning in coronavirus infections

### SARS-CoV

**Fig. 1:**
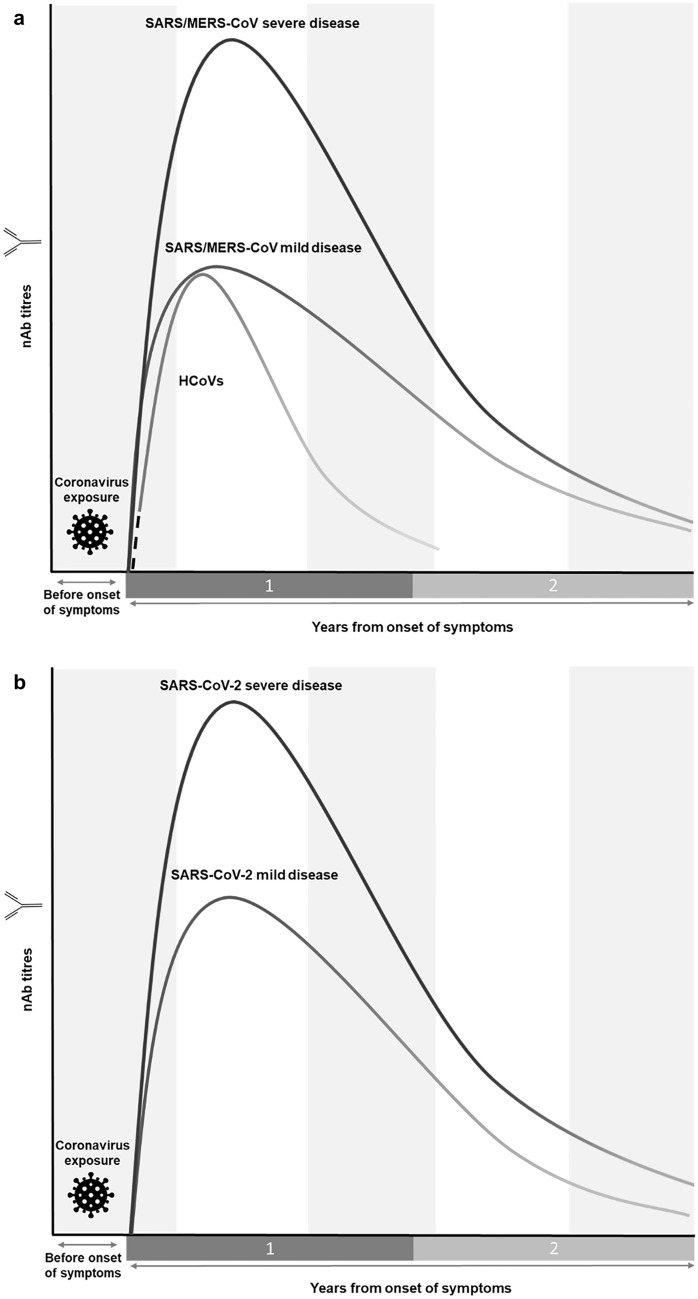
Graphical representation of the longevity and magnitude of the nAb antibody response to coronaviruses. **a** Shows trends in antibody kinetics to SARS-CoV, MERS-CoV and HCoVs, highlighting the relatively rapid waning of HCoV nAbs as well as higher titres generated in severe SARS-CoV/MERS-CoV infection [28–32, 34–38, 40–42, 44, 52]. The dotted line indicates a lack of serological data for common cold coronavirus infections in individuals naïve to the infection. **b** Compares antibody titre trends in severe and mild SARS-CoV-2 and their waning over time, highlighting the higher titres generated in severe infection [56–58, 60, 62–64]. Neither graph drawn to scale. *SARS-CoV* severe acute respiratory syndrome coronavirus, *MERS-CoV* Middle East respiratory syndrome coronavirus, *HCoV* human coronavirus

IgM antibodies reach peak titres ~ 1 month post-symptom onset [28–30]. whereas IgG and nAb reach theirs ~ 2–4 months [28–32] and 1–4 months [29, 31], respectively. Subsequent titres of IgM begin a relatively rapid decline, decreasing steadily to undetectable levels ~ 6 months post-symptom onset [29, 32]. IgG and nAb display a more gradual and closely correlated pattern in their waning, approaching values for seronegativity ~ 2 years post-symptom onset (Fig. 1a) [28, 29, 31]. At ~ 3 years, close to half of initially IgG positive patients revert to seronegative status [29] and by 6 years, almost all patients revert to IgG seronegativity to SARS-CoV [33].

Higher titres of nAb are positively correlated with symptomatic and more severe clinical disease [34–36] but whether the severity of disease affects subsequent antibody waning is not clear, with conflicting results found in different studies [31, 34, 37]. The presence of underlying comorbidities, age and steroid use does not appear to be associated with different antibody kinetics [31, 37], though it has been noted that men exhibit a more pronounced decrease in nAb titres compared to women [31, 34].

### MERS-CoV

Robust antibody responses to MERS-CoV develop by week 3 [38–41]. IgG titres decline during weeks 4–5 and though the IgM titres start decreasing earlier, they are seropositive for > 1 month, albeit at a lower titre than IgG [39]. Observations have been made that while a more severe disease is associated with higher antibody titre peaks [38, 42–44], a delayed nAb response has been observed [40, 42]. Although antibody waning occurs, IgG and nAbs are detectable > 1 year post-symptom onset [41, 43–45], with cases of antibody persistence for up to 34 months in recovered individuals [45]. Importantly, antibodies wane at a slower rate during months 6–12 compared to the first 6 months post-symptom onset (Fig. 1a) [44]. Additionally, antibody response longevity correlates with disease severity [43, 44] such that most patients with severe disease have detectable IgG and nAb after 1 year compared to 33% of individuals who experienced mild disease [44]. On the other hand, age does not seem to be correlated with nAb response [46], however, few studies have investigated this factor.

### HCoVs

Antibodies to HCoV infections may be protective but wane quickly. Seroepidemiological studies have shown HCoV IgM to be present in children but absent in adults, indicating that first infection occurs during childhood [47]. The majority of seroconversion is reported to occur before the age of 3.5 years [48]. Persistence of antibodies in the adult population is likely related to frequent reinfection [49–51]. Experimental infection with HCoV-229E has shown that peaks of total IgM, IgG and nAb titres occur 12–14 days after inoculation, falling considerably by 12 weeks and to near baseline levels by 52 weeks (Fig. 1a) [52]. However, unlike serological studies of SARS-CoV, SARS-CoV-2 and MERS-CoV, where patients are most likely naïve to the infection, population seropositivity to HCoVs is high which affects the conclusions which can be drawn from human challenge studies [53]. In one study, antibodies to the four HCoVs were detectable in > 70% of the adult population [48]. Reinfections after challenge may not be due to a lack of immunity but rather due to the unusually high inoculum dose [54].

### SARS-CoV-2

In a SARS-CoV-2 study, three different patterns of seroconversion have been observed. In some, IgM appears before IgG as expected, in others they occur simultaneously and sometimes IgM appears after IgG [5]. Overall, IgM, IgG and nAb titres peak ~ 2–3 weeks post-symptom onset and decline to undetectable levels by 6 weeks for IgM, whereas IgG and nAb reach a plateau before declining within 2–3 months (Fig. 1b) [6, 55–57]. Mathematical modelling estimates that within 1 year IgG antibodies to nucleocapsid, spike protein and RBD wane to 7%, 36% and 31% of their titres at 2 weeks post-symptom onset, respectively [58]. Additionally, nAb responses seem to correlate with disease severity [7, 56, 59], with antibody half-lives of 31 and 69 days in asymptomatic and severe infections, respectively [60].

IgG titres to SARS-CoV-2 infection are negatively correlated with age for those < 18 years but positively correlated with age in adults [61]. Within 6 months post-symptom onset, older adults (44–66-year-olds) seem to maintain higher IgG levels than younger adults (18- to 44-year-olds) but no difference is observed at 12 months post-symptom onset [62]. In children, the narrower breadth of anti-SARS-CoV-2-specific antibodies, specifically with reduced generation of anti-nucleocapsid IgG and nAb compared to adults, has been associated with a milder disease course [63].

## Implications of waning antibodies on COVID-19

### Underlying mechanisms of antibody waning

In SARS-CoV-2 the initial rapid waning of antibodies is thought to be due to the loss of short-lived plasma cells, while the plateau in antibody levels occurs due to establishment of long-lived plasma cells [65]. The underlying causes of waning were investigated in a recent paper by Kaneko et al*.*, which found the absence of germinal centres in the thoracic lymph nodes of deceased SARS-CoV-2 patients [66]. They proposed this lack of germinal centres was due to defective Bcl6+ follicular T-cells, which are unable to activate memory B-cells (MBCs). In turn this would impair the production of long-lasting and high-affinity antibodies, which could explain the rapid waning of antibodies in SARS-CoV-2 [66]. A similar mechanism for rapid waning of antibodies was proposed in SARS-CoV, where it was found that the virus depleted key lymphocytes involved in immune signalling and affected germinal centre responses [67]. However, since both studies were done on deceased patients, these mechanisms only explain waning in the most severe cases.

### Duration of antibody-mediated immunity

Time to reinfection can help determine the duration of protective immunity. Unlike other coronaviruses, reinfections with HCoVs have been widely observed. These usually occur within 12 months of the preceding infection, though some manifest as early as 6 months with no association with waning antibodies [52, 53, 68]. It is important to note that reinfection with HCoVs may be associated with less severe disease and a shorter duration of shedding, but results have been contradicting [51, 52, 69].

Furthermore, a lack of genotypic difference between reinfecting HCoV-NL63 strains has been confirmed which means mutations may not be responsible for reinfections and therefore, antibody-mediated immunity to HCoVs is short-lasting if at all protective [70]. In two rhesus macaque trials, previous SARS-CoV-2 infection was protective against reinfection when re-exposed at 28 and 35 days, showing greater nAb titre production upon re-challenge in comparison to primary challenge [71, 72]. While SARS-CoV-2 reinfection cases are rare, they have occurred, with one study reporting a reinfection rate of 0.02% and median time to reinfection of 64.5 days [73]; this is shorter than what is seen with HCoVs, suggesting a relatively short period of protective immunity. However, this study may have overestimated the reinfection rate due to diagnostic error and a small sample size [73].

A correlation between severity of illness and magnitude of humoral response in MERS-CoV, SARS-CoV and SARS-CoV-2 has been reported [7, 34–36, 43, 59]. In the case of SARS-CoV and MERS-CoV, this has been associated with a longer time to seronegativity, but results have been conflicting [20, 23, 26, 33, 34]. Therefore, whether severe cases of SARS-CoV-2 will have longer lasting immunity remains to be confirmed.

### Efficacy of antibodies

The efficacy of antibodies is a crucial aspect of immunity. Some studies have suggested that antibodies are not sufficient for viral clearance [74]; this is supported by the absence of an abrupt decline in viral load after seroconversion [75]. One way of assessing the efficacy of antibodies may be through the observation of patient response to convalescent plasma transfer therapy (CPTT). Studies of CPTT in SARS-CoV-2 have shown varied results on the protective role of nAbs. Initially, CPTT demonstrated encouraging results in case–control studies for severe SARS-CoV-2 [76, 77], with some studies proposing earlier therapy being more beneficial [77, 78]. Early CPTT was also found to be beneficial in SARS-CoV [79]. However, recently published data from large-scale randomised controlled trials did not identify any significant reduction in mortality or improvements in clinical outcomes for those with mild or severe SARS-CoV-2 receiving CPTT [80, 81]. This suggests that the antibody response alone may not be as important as once thought in SARS-CoV-2 immunity.

In cases of recovered COVID-19, assessing the efficacy of antibodies against reinfection is difficult. For example, a large COVID-19 outbreak on a Seattle fishery vessel infecting over 85% of the crew on board showed that those who were positive for nAbs (titres ranging from 1:161 to 1:3082), prior to departure successfully remained infection-free [82]. However, this correlation of antibodies and protection does not necessarily imply a causative relationship.

Previous in vivo and in vitro studies with MERS-CoV and SARS-CoV have cautioned of antibody-dependent enhancement (ADE) in SARS-CoV-2 [83]. While ADE has not been noted in COVID-19 patients so far, preliminary findings from an in vitro analysis of COVID-19 convalescent plasma identified a significantly greater likelihood of ADE for patients who were older, had a more severe infection and a longer disease duration [84]. ADE was greatest in plasma with high titres of SARS-CoV-2-specific anti-RBD and anti-S1 antibodies [84]. Importantly, cross-reactive antibodies from other coronaviruses were excluded as the cause of ADE [84]. While the mechanism of ADE here is not clear, it could suggest a less efficacious antibody response in certain cohorts.

### B- and T-cell immunity

In 10–30% of recovered COVID-19 cases, antibody titres are low or undetectable [85, 86]. Therefore, other aspects of humoral immunity are likely at play [87, 88]. For example, MBCs are thought to be maintained independently of antibody levels, which means B-cell immunity may persist even if antibodies wane [89]. Though, it has been noted that MBCs in SARS-CoV are undetectable 6 years after infection [33]. In SARS-CoV-2, MBCs (specific to spike and nucleocapsid proteins) and memory T-cells (MTCs) have been shown to persist for at least 3 months when antibody levels decline, but follow-up has been limited due to the ongoing pandemic [87, 88].

Promisingly, MTCs in MERS-CoV and SARS-CoV have been shown to persist for 10 years [90] and 17 years [91], respectively, which shows potential for long-lasting immunity against SARS-CoV-2. However, whether T-cells can form protective immunity without an antibody response is still uncertain and cannot be deduced from SARS-CoV, as this no longer circulates to cause reinfection [15, 92]. Furthermore, levels of IgG and IgA have been shown to correlate with the number of specific CD4^+^ T-cells, therefore it may be that T-cells can wane in a similar manner to antibodies [93].

It is known that cellular immunity is important in protection against viral infection, given that children without it have worse outcomes than those with low or absent antibody titres in conditions such as hypogammaglobulinaemia or agammaglobulinaemia [94]. Additionally, a report of COVID-19 in two patients with agammaglobulinaemia showed recovery without severe disease suggesting T-cells may be more important in overcoming SARS-CoV-2 infection than B-cells [95]. The importance of T-cells has also been highlighted in studies showing worse COVID-19 outcomes in HIV patients not on antiretroviral therapy (ART) compared to those on ART; worse outcomes potentially being attributed to increased T-cell exhaustion in these patients [96, 97]. However, reports of reinfection or possible persistence of SARS-CoV-2 in patients on B-cell depleting immunosuppressants, e.g. rituximab, which prevent the generation of an antibody response to SARS-CoV-2, suggest that antibodies are likely to be vital in protection against reinfection [98, 99].

### Cross-reactivity and trained immunity

In serological studies of SARS-CoV-2 it is widely assumed that the antibody response mounted is against a novel virus. However, HCoV cross-reactivity may be affecting the antibody dynamics [100] and it has been suggested to be the reason for lower disease severity in children and lower death rates in low- and middle-income countries [101, 102]. Nevertheless, in vitro, pre-existing cross-reactive antibodies were not protective against SARS-CoV-2 infection of Vero E6 cells [100].

Some have suggested a possible anamnestic response in SARS-CoV-2 from pre-existing MBCs. A study [103] identified that > 80% of low-affinity antibodies which cross-reacted to SARS-CoV and SARS-CoV-2 also reacted to spike protein components of HCoV. These cross-reactive antibodies had higher levels of clonal expansion than those which only reacted to SARS-CoV and SARS-CoV-2, possibly suggesting a boosted response from pre-existing MBCs [103]. This cross-reactivity may mean that initial immunity for SARS-CoV-2 is higher than expected, which could have positive implications for herd immunity [104]. Furthermore, when examining cross-reactivity, the observed results may be due to defective assays and more studies need to make the distinction between cross-reactivity and cross-binding [105].

A pre-existing nAb response has also been noted in MERS-CoV and SARS-CoV-2 vaccine clinical trials [106, 107], likely due to antibody cross-reactivity with HCoVs. However, in both groups this did not alter the vaccine immunogenicity profile or subsequent antibody dynamics, indicating SARS-CoV-2 vaccinations would not have reduced immunogenicity despite interactions with other seasonal coronaviruses. This has positive implications in the scenario where SARS-CoV-2 outbreaks become a yearly phenomenon.

Innate immunity can also be “trained” using vaccines such as Bacillus Calmette–Guérin (BCG) and microbial elements, e.g. lipopolysaccharides, inducing epigenetic and metabolic changes in myeloid cells [108]. BCG vaccinations have previously been shown to be protective through the enhancement of antibody release in influenza A (H1N1), reduction in clinical manifestations of herpes simplex virus infections and the decrease in yellow fever vaccine viraemia [109]. Recent epidemiological studies suggest that BCG vaccination may be protective against severe COVID-19 [110], though this may be affected by various confounding factors. Clinical trials to confirm potential benefits in response to SARS-CoV-2 are ongoing, e.g. NCT04659941, NCT04537663 and NCT04327206.

### Vaccines

#### Viral vector vaccines

The relatively rapid resolution of SARS-CoV and MERS-CoV, and lack of interest in HCoV research has resulted in limited vaccination experience for coronaviruses [111]. However, the ChAdOx1 MERS trial has guided much of the current approach to the ChAdOx1 nCoV-19 vaccine [106, 107]. Similarly to ChAdOx1 MERS, ChAdOx1 nCoV-19 produced a strong IgG and nAb response, with a peak in antibody titres by day 28 that remained elevated at day 56 [106, 107, 112]. In the ChAdOx1 MERS trial, waning of antibodies continued to day 182, though levels plateaued after this point, remaining detectable even at the end of the 346-day follow-up period [106]. However, it is unclear if such low titres are protective against infection.

Furthermore, antibodies to the viral vector ChAdOx1 have the potential to impact vaccine efficacy, hence the use of a simian virus with rare pre-existing immunity in the aforementioned trials [113, 114]. After prime vaccination in the ChAdOx1 nCoV-19 trial, anti-ChAdOx1 nAb increased in both low and standard dosages, peaking by day 28. Antibodies plateaued at this level even after the booster dose until the end of follow-up at day 56 [112]. Studies have noted that low levels of pre-existing nAb to simian adenovirus vectors do not reduce the vaccine-induced immunological response [114, 115]. However, higher levels of vector nAb triggered by prime vaccination may interfere with subsequent booster doses, as indicated in the phase II/III ChAdOx1 nCoV-19 trial which noted a weak inverse correlation between anti-ChAdOx1 nAb and anti-spike IgG [112]. This might explain the greater efficacy of the ChAdOx1 nCoV-19 vaccine with greater interval between the priming and booster doses [84, 116].

In the case of Sputnik-V, the heterologous combination of the rAd26-S and rAd5-S adenovirus vectors mitigated the issue of primer-induced anti-vector antibodies and could be why a higher efficacy of 91.6% (95% confidence interval, CI 85.6–95.2%) was seen [117] compared to the 66.7% (95% CI 57.4–74.0%) of ChAdOx1 nCoV-19 [116] at preventing symptomatic COVID-19. Importantly, models have predicted that as variants arise which are less susceptible to pre-existing vaccine-induced nAb, vaccines with higher initial efficacy against the wild-type would similarly provide higher efficacy against variants [112, 118].

#### mRNA vaccines

Despite their novelty, the BNT162b2 and mRNA-1273 mRNA vaccines have successfully demonstrated robust ability at generating nAb titres at levels superior to most other vaccines, including viral vector approaches [118]. Expectedly, the increased titre correlates with increased vaccine-mediated protection from COVID-19 of 95% (95% CI 90.3–97.6%) and 94.1% (95% CI 89.3–96.8%) for the BNT162b2 and mRNA-1273 vaccines, respectively [118–120]. Similarly to ChAdOx1 nCoV-19, increased intervals between primer and booster mRNA vaccine doses have yielded superior nAb titres. However, a study by Parry et al. noted that this may come at the cost of a reduced cellular immune response [121]. Additionally, an age-related decrease in mRNA vaccine-induced antibodies and cellular responses were noted by several studies [122–126], with some reporting faster waning in older age groups [122]. Therefore, additional investigation on the impact of dosing schedules for different age groups is warranted. Waning of mRNA vaccine-induced nAb responses have also been reported as early as 6 weeks post-booster dose, continuing at 12 weeks [122], but longer follow-up is crucial.

#### Natural infection and vaccination

Vaccines generally induce comparable or greater antibody titres against SARS-CoV-2 than natural infection, which is differentiated from vaccine-acquired immunity by the presence of anti-nucleocapsid antibodies. Three weeks after a single dose of the BNT162b2 vaccine, comparable anti-spike IgG titres to convalescent patients are induced, rising significantly 1 week after the subsequent booster dose [122]. Similarly, nAb levels induced after complete regimens of NVX-CoV2373, mRNA-1273 and Sputnik-V have all shown higher nAb titres than convalescent samples [118]. Results of ChAdOx1 nCoV-19 trials have shown nAb titres near or below convalescent patients, corresponding to the lower protection offered than from some other vaccines [107, 118].

Single-dose vaccination of patients with previous SARS-CoV-2 exposure has been found to induce dramatic increases in titres of anti-spike IgG and nAbs [127], rivalling titres generated after booster doses in infection-naïve subjects [128, 129]. Antibody responses after a single vaccine dose in those previously infected develop quicker and reach higher titres [123], a phenomenon occurring even when anti-spike IgG from previous SARS-CoV-2 infection had waned to low or undetectable levels [124], indicating immune memory despite waning of antibodies. This may be important in rationing vaccines [125], especially considering ongoing shortages around the world. However, despite the enhanced peak antibody response following vaccination post-infection, the subsequent 8 weeks of follow-up have shown a faster decline in antibody titres compared to infection-naïve vaccinated patients. Therefore, longer follow-up is needed to see if a higher plateau is finally reached [121].

#### Vaccine mixing

Studies into the immunogenicity of heterologous prime-boost vaccination using the ChAdOx1 nCoV-19 and BNT162b2 vaccines have been initiated. This combination of vaccines generated a stronger antibody response [126] than two doses of the ChAdOx1 nCoV-19 vaccine, likely as the neutralising effects of anti-vector nAbs were avoided, as with Sputnik-V [117, 130], although a comparison to two doses of the BNT162b2 vaccine is yet to be done.

Despite the positive antibody response with vaccine combinations, preliminary data suggest that mild–moderate side effects increase in frequency with mixed vaccines compared to two doses of the same vaccine [131]. All in all, further research is needed to determine the best vaccine regimen for long-term protection against SARS-CoV-2.

### Herd immunity and SARS-CoV-2 variants

For SARS-CoV-2, it was initially estimated that at least 50–66.7% of the population needs to be immune [132] in order to achieve herd immunity. Assuming this level of immunity can be reached, the length of time and effectiveness of the immune response is an important consideration [132], as transient immunity from antibody waning would mean COVID-19 outbreaks could become biennial or annual [104]. Achieving this through natural infection is unlikely to be a viable option due to unacceptably high morbidity and mortality rates. This was evident in an uncontrolled outbreak of SARS-CoV-2 infection in the Amazon area of Manaus, which experienced a 4.5-fold increase in excess deaths when three quarters of the population were infected [133]. Theoretically, this should have been enough for herd immunity (> 67%) but Manaus unexpectedly experienced a second resurgence in January 2021 [134], just months after the first peak in June 2020 despite high seropositivity. Although other epidemiological studies show that naturally acquired immunity should be as protective against reinfection as vaccination for at least 5 months [135], achieving herd immunity through vaccinations appears more desirable.

The percentage required for herd immunity may be an underestimation due to under-reporting of cases [132] and the potentially lower transmissibility [136] of infection in children. Moreover, mutations conferring greater transmissibility, such as N501Y (found in B.1.1.7 and B.1.351 variants), E484K (found in the B.1.351 variant), and L452R and E484Q (found in the B.1.617.2 variant), increase the basic reproduction number [137–139] which in turn increases the percentage needed to achieve herd immunity [132].

Based on early results from in vitro studies of recovered SARS-CoV-2 patients’ CPTT and neutralising monoclonal antibodies, it appears these mutations may result in decreased effectiveness of pre-existing antibodies [140]. Despite this possibility, recently a study [141] published that the B.1.1.7 variant has no significant impact on vaccine-induced immunity. Comparing the B.1.617.2 variant to the B.1.1.7 variant, only a small decrease in vaccine effectiveness 2 weeks after the second dose was seen for both the BNT162b2 (93.4% to 87.9%) and ChAdOx1 nCoV-19 (66.1% to 59.8%) vaccines [142]. However, a decrease in effectiveness of either vaccine against B.1.617.2 was significantly more pronounced after only a single dose [142], stressing the importance of vaccine regimen completion. In addition, although the neutralisation titres reduce by 6.4-fold for the B.1.351 variant, the titres remain high with the ability to neutralise pseudoviruses [140].

Interestingly, mRNA vaccines induce disproportionately more anti-RBD antibodies compared to natural infection, which tend to target other portions of the spike protein [143]. Vaccine-induced antibodies also target a broader range of areas on the RBD, meaning those antibodies are better able to respond potently against new variants even when they carry mutations in the RBD [143]. Therefore, while the emergence of new SARS-CoV-2 variants remains a threat to herd immunity, current vaccines remain effective and provide superior protection to natural disease.

Vaccine hesitancy, however, is a major challenge to achieving herd immunity and modelling suggests that countries with lower vaccine uptake may experience eightfold greater deaths over a 2-year period [144]. Promisingly, a recent study by Milman et al. has found that high levels of vaccine uptake reduce transmission of SARS-CoV-2 even in the unvaccinated cohorts [145], which may curb the pandemic. Given the presence of unvaccinated individuals, varying levels of immunological protection from vaccination/natural infection, potentially lower efficacy of vaccines against new variants and reports of reinfection, low-level transmission is expected to continue after the end of the pandemic. However, severity of these infections is likely to be much lower even with low nAb titres [118]. Endemic circulation such as that of HCoVs, which probably caused similar pandemics in the past, will likely maintain population immunity against SARS-CoV-2 [146]. This may eliminate the need for booster vaccinations.

## Limitations and future

Given the low case fatality seen in HCoVs as well as the relatively small outbreaks of SARS-CoV and MERS-CoV, prior research into coronaviruses has been lacking. Additionally, the most genetically homologous coronavirus, SARS-CoV, no longer circulates [15] which puts limits on what we can deduce about the long-term efficacy of the immune response against it. Moreover, due to the fast-evolving nature and high volume of scientific publishing on COVID-19, this review may lack inclusion of more recent studies.

Studies have also noted that antibodies specific to certain viral antigen, such as nucleocapsid or spike protein may wane at different rates [147–149], though it is unclear to what extent this may be due to inaccuracies in the serological method employed. The limited research addressing antibody targets for SARS-CoV, MERS-CoV and HCoVs makes it difficult to contextualise their importance. Enzyme-linked immunosorbent assay (ELISA) is one of the most frequently used assays in determining antibody-specific IgG titres though substantial variations exist in its sensitivity and specificity [150]. Furthermore, while the plaque reduction neutralisation test (PRNT) is considered the “gold standard” [12, 151] in assessing the functional ability of antibodies in viral neutralisation, other simpler assays have frequently been used. These generally correlate well to PRNT [12], though this heterogeneity of platforms remains an important caveat when comparing results.

A further limitation of the current body of research is the lack of focus on mucosal immunity and the waning of secretory IgA. Secretory IgA is known to have a crucially protective function at the mucosal surface [152] and is possibly an even more potent neutraliser of SARS-CoV-2 than IgG [153]. If further research into this proves fruitful, the mucosal route of vaccine delivery could be of greater interest.

Existing literature shows that antibodies to coronavirus infections wane over time but is difficult to quantify what antibody titre conveys protection from SARS-CoV-2 and for how long titres can be maintained above this threshold. To address these points, animal re-challenge studies of SARS-CoV-2 could be initiated with a longer time interval to reinfection, as only a short-time window of ~ 30 days has been tested [71, 72].

## Conclusion

The last 20 years have taught us that coronaviruses have immense pandemic potential and should be monitored carefully. By reviewing the literature on SARS-CoV, MERS-CoV and HCoVs, we have concluded that high antibody titres to SARS-CoV-2 are unlikely to be maintained in the long-term; antibodies to most coronaviruses wane to undetectable titres within 2 years of infection and within 6–12 months following HCoV infections. Furthermore, although various vaccine platforms have proven their ability to induce robust antibody responses, this is accompanied by subsequent waning, making reinfection a possibility unless “booster” doses are administered. Therefore, public health measures relying on the induction and monitoring of antibodies for herd immunity should be considered carefully. Nevertheless, there is evidence that B- and T-cells persist for longer than antibodies and vaccines targeting these may be a promising strategy for long-term immunity. However, these also require further research to determine their protective capacity.

## Data Availability

All data published were publicly available.

## Abbreviations

COVID-19: Coronavirus disease 2019
SARS-CoV-2: Severe acute respiratory syndrome coronavirus 2
SARS-CoV: Severe acute respiratory syndrome coronavirus
MERS-CoV: Middle East respiratory syndrome coronavirus
HCoVs: Human coronaviruses (HCoV-229E, HCoV-OC43, HCoV-NL63, HCoV-HKU1)
nAb: Neutralising antibody
RBD: Receptor-binding domain
MBC: Memory B-cell
MTC: Memory T-cell
ART: Antiretroviral therapy
BCG: Bacillus Calmette–Guérin
CPTT: Convalescent plasma transfer therapy
ADE: Antibody-dependent enhancement
ELISA: Enzyme-linked immunosorbent assay
PRNT: Plaque reduction neutralisation test
